# Sex Categorization of Faces: The Effects of Age and Experience

**DOI:** 10.1177/2041669519830414

**Published:** 2019-02-28

**Authors:** Anne Hillairet de Boisferon, Eve Dupierrix, Lesley Uttley, Lisa M. DeBruine, Benedict C. Jones, Olivier Pascalis

**Affiliations:** Laboratoire Vision Action Cognition, Universite Paris Descartes, Boulogne-Billancourt, France; Laboratoire de Psychologie et NeuroCognition, Grenoble, France; University of Sheffield, UK; Institute of Neuroscience and Psychology, University of Glasgow, UK; Laboratoire de Psychologie et NeuroCognition, France

**Keywords:** experience, face categorization, own-age bias, sex discrimination

## Abstract

The face own-age bias effect refers to the better ability to recognize the face from one's own age compared with other age groups. Here we examined whether an own-age advantage occurs for faces sex categorization. We examined 7- and 9-year-olds' and adults' ability to correctly categorize the sex of 7- and 9-year-olds and adult faces without external cues, such as hair. Results indicated that all ages easily classify the sex of adult faces. They succeeded in classifying the sex of child faces, but their performance was poorer than for adult faces. In adults, processing time increased, and a response bias (*male* response) was elicited for child faces. In children, response times remained constant, and no bias was observed. Experience with specific category of faces seems to offer some advantage in speed of processing. Overall, sex categorization is more challenging for child than for adult faces due to their reduced sexual dimorphic facial characteristics.

## Introduction

The way we interact with individuals is based on different social attributes such as age and gender. Those attributes can be provided by faces and are used from an early age (Quinn, Yahr, Kuhn, Slater, & Pascalis, 2002). One of the most salient social categories conveyed by human faces is sex.

Many sexually dimorphic features in faces may underpin sex categorization: the brows, eyes, the whole jaw, the chin, the nose, and the mouth (Brown & Perrett, 1993; Burton, Bruce, & Dench, 1993; Chronicle et al., 1995). Configural relationships among individual features also influence sex classification (Brown & Perrett, 1993). Adults and children can rapidly and accurately determine the sex of adult faces (Burton et al., 1993; Wild et al., 2000). Moreover, infants begin to discriminate human faces based on sex information between 3 and 12 months of age (Leinbach & Fagot, 1993; Quinn et al., 2002). Many studies however reported an asymmetrical learning of female versus male faces and suggested that differential experience with faces may be at the origin of better categorization for female versus male faces (see Ramsey, Langlois, & Marti, 2005, for a review). Many other lines of evidence suggest that extensive experience with a category of faces may influence its processing (e.g., Kuefner, Macchi Cassia, Vescovo, & Picozzi, 2010; McKone, Brewer, MacPherson, Rhodes, & Hayward, 2007). Of greatest relevance for our study, an own-age bias (OAB) in face recognition has been observed. Younger adults recognize young adult faces better than older adult faces (He, Ebner, & Johnson, 2011), child and neonate faces (Kuefner, Macchi Cassia, Picozzi, & Bricolo, 2008), or infant faces (Chance, Goldstein, & Andersen, 1986). Anastasi and Rhodes (2005) found that both children and adults recognize better own-age faces. Own-age bias however happens to be modulated by experience, maternity nurses (Macchi Cassia, Picozzi, Kuefner, & Casati, 2009), and school teachers (Kuefner et al., 2008) are better at recognizing neonate and child faces than are participants with less experience with these face categories. Similarly, nursing home assistants were equally proficient at recognizing younger and older adult faces, whereas adults with limited contact with elderly individuals were better at recognizing younger adult faces (Proietti, Pisacane, & Macchi Cassia, 2013). These findings suggest that the OAB is likely to reflect the predominant age of the faces present in an individual's environment, or in other words, that the amount of experience with other-age faces is likely correlated with the magnitude of the OAB (for a review and meta-analysis, see Rhodes & Anastasi, 2012). The fact that individuals have more extensive experience, but not always, with members of their own age-group, can explain the following pattern of results: Young children with siblings showed similar recognition abilities for adult, infant, or child faces, whereas young children without siblings recognized better adult faces (Macchi Cassia et al., 2009; Macchi Cassia, Pisacane, & Gava, 2012; Macchi Cassia, Proietti, & Pisacane, 2013).

Experience with a given face category can also modulate face categorization, such as sex categorization. O'Toole, Peterson, and Deffenbacher (1996) found an own-race advantage when adult participants were asked to categorize the sex of own- and other-race faces. Similarly, 3-month-olds no longer showed a preference for female faces when presented with other-race faces (Quinn et al., 2008).

Wild et al. (2000) found that, whereas both adults and children can accurately judge the sex of adult faces, performance drops markedly when asked to judge the sex of child faces. Their youngest participants, aged 7 years, were even unable to categorize the sex of child faces. These results are surprising given that experience with a category of faces enhances sex-categorization performance (O'Toole et al., 1996). As children grow older, their exposure to peers' faces increases and should improve their ability to determine the sex of a person. Wild et al. (2000) used, however, only a small sample of children's faces between 7 and 10 years of age instead of faces that were the same age as their participants. It is thus difficult to determine if the children's poor performance was due to a genuine difficulty in sex categorization of other child faces or to unfamiliarity with the age categories of the child faces used, 7-year-olds having to judge the gender of 9-year-olds, and vice versa.

In the current study, we investigated sex categorization of faces in the same age groups as Wild et al. (2000) study (adults, 7-year-olds, and 9-year-olds), using face images of unfamiliar individuals belonging to the same category of age as participants. We hypothesized that all age groups would be better at categorizing the sex of adult faces than child faces (Hypothesis 1) because of their greater experience with adult faces compare to children faces but also because sexually facial characteristics are more pronounced in adults. Second, we also expected an own-age advantage that will interfere with the large advantage for adult faces. We predicted a drop in sex-categorization performance for child faces, regardless of their age, compared with adult faces that would be more pronounced for adult participants than for child participants. In other words, only child participants should benefit from their greater experience with child faces and demonstrate a drop in magnitude of the *adult bias* in sex categorization of faces (Hypothesis 2). Indeed, as children grow older, especially when children enter school, they continue to be exposed to other adult face, but they are mostly exposed to other child faces. Finally, if there is a *specific* own-age advantage in children's sex categorization of child faces, we also hypothesized that children would be better at categorizing the sex of their exact age peer faces (Hypothesis 3).

## Method

### Participants

Fifteen adults (6 males, mean age = 27.1 years, age range = 20–51 years), eighteen 9-year-olds (8 males, mean age = 9.3 years, age range = 9.0–10.4 years), and eighteen 7-year-olds (9 males, mean age = 7.8 years, age range = 7.3–8.2 years) participated in the study. All participants were Caucasian.

### Stimuli

**Figure 1.**
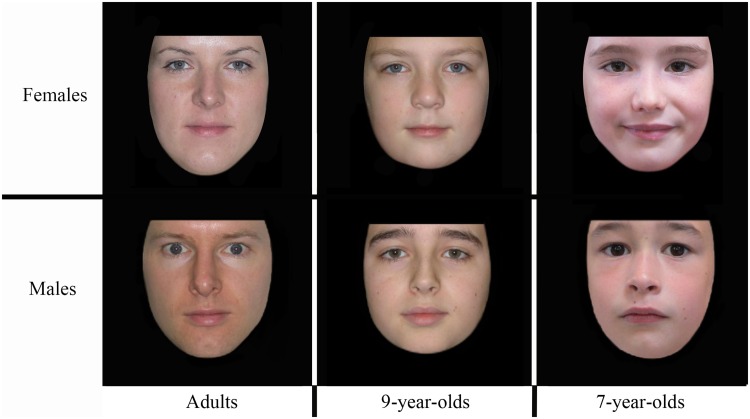
Examples of male and female stimuli for each age of faces condition.

Twenty different images of unfamiliar Caucasian faces (10 males and 10 females), with neutral expression and direct gaze, were used in each age category (adults, 9-year-olds, and 7-year-olds). All faces were masked at the outline of the face, and the position of the pupils was normalized. All hair cues were masked; only the lower face shape and internal features were visible. The stimuli were approximately 12 cm × 12 cm when presented on the screen (Figure 1).

### Procedure

Participants were seated in front of a laptop computer and instructed that they will have to decide whether they believed each face to be male or female. Their responses were recorded via the computer keyboard. Participants were asked to respond as fast as possible but were informed that accuracy was more important than speed of response. Correct responses and reaction times are presented in Table 1.

**Table 1.** Mean Number of Correct Responses and Reaction Times (±*SD*s) for Sex Categorization of All Age and Sex of Faces for All Participant Age Groups.

		Age of faces		
	Age groups	7-year-olds	9-year-olds	Adults
Correct responses	7-year-olds	11.1 (2.3)	12.7 (2.0)	18.6 (1.2)
	9-year-olds	11.6 (2.3)	12.1 (2.6)	18.5 (1.4)
	Adults	13.2 (1.4)	12.9 (2.8)	19.4 (0.9)
Reaction times (ms)	7-year-olds	1,374 (347)	1,347 (458)	1,242 (309)
	9-year-olds	1,251 (385)	1,151 (341)	1,090 (263)
	Adults	1,454 (415)	1,189 (363)	860 (184)

All participants completed all three experimental conditions (adult stimuli, 9-year-old stimuli, and 7-year-old stimuli) in the following order: The adult condition first, since categorizing the sex of adult faces is known to be easily accomplished (Burton et al., 1993; Wild et al., 2000) and was suitable for familiarizing participants with the experimental procedure. The 9-year-old and 7-year-old face conditions were done in a counterbalanced order. Within each condition, trial order was fully randomized.

## Results

### Data Reduction

We removed from our analyses responses for which response times (RTs) were under 500 ms and more than two standard deviations from participants' mean RTs in each age of faces condition (loss of 3.2% of the responses).

### Correct Responses

Sex classification accuracy was measured with *A*′, a nonparametric measures of sensitivity based on signal detection theory that control for response bias (Stanislaw & Todorov, 1999). *A*′ is computed based on hit (*H*) rate and false alarm (*F*) rate, as follows: *A*′ = 1/2 + (*H*−*F*) (1 + *H*−*F*) / [4*H* (1−*F*)], when hits (*H*) > false alarms (*F*), and, *A*′ = 1/2 + (*F*−*H*) (1 + *F*−*H*) / [4*F* (1−*H*)], when hits (*H*) < false alarms (*F*).The hit rate is computed by dividing the number of hits by the total number of signal trials. Similarly, the false-alarm rate is computed by dividing the number of false alarms by the total number of noise trials. *Hits* are defined as the response *female* to female faces, and *false alarms* defined as the response *female* to male faces. An *A*′ score of .50 corresponds to chance performance.

We also computed a response bias measure (*C*), that reflect either the degree to which *male* responses are preferred (positive bias), or the degree to which *female* responses are preferred (negative bias). *C* is computed as follows: *C* = −l/2 [*z*(*H*) + *z*(*F*)]. A zero value indicates no bias.

### Accuracy

We first tested sex classification performance against chance (i.e., .50) by performing one-sample *t* tests separately. Analyses revealed that all participant groups categorized the sex of the faces in each of the three experimental conditions at levels above chance (all *t*s > 6.32, all *p*s < .001, all Cohen's *d* > 1.63).

Preliminary analyses showed no significant differences between male and female participants in sex categorization of faces and revealed no interactions with the sex and age of the faces factors. Therefore, the data were collapsed across sex of the participants.

**Figure 2.**
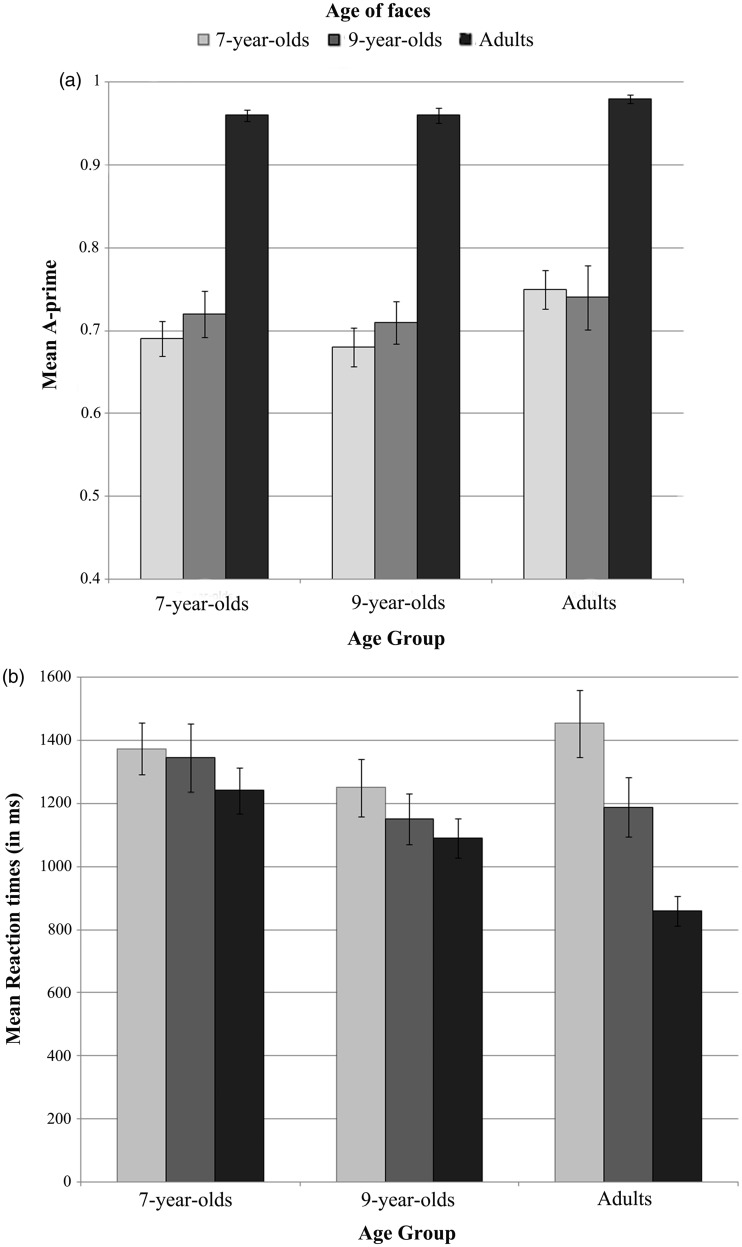
(a) Mean *A*′ scores (±*SE*s) and (b) mean RTs (±*SE*s) for sex categorization of all age of faces for all participant age groups.

Accuracy data (Figure 2) were analyzed using a two-way mixed analysis of variance (ANOVA) with *age of participants* (7-year-olds, 9-year-olds, and adults) as a between-subjects factor and *age of faces* (7-year-olds, 9-year-olds, and adults) as a within-subject factor. This analysis revealed only a significant main effect of the *age of faces*, Greenhouse–Geisser adjusted *F*(1.69, 81.27) = 138.7, *p* < .001, $\eta_{\text{p}}^{2}=.74$. Mean *A*′ scores were better for adult faces (*M* = .97, *SD* = .04) compared with both 7-year-old faces (*M* = .70, *SD* = .10) and 9-year-old faces (*M* = .72, *SD* = .13), that did not differ from each other. The main effect of the *age of participants, F*(2, 48) = 2.14, *p* = .13, $\eta_{\text{p}}^{2}=.08$, and the interaction between *age of faces* and *age of participants, F* < 1, were not significant.

We performed a priori contrasts to test our specific hypotheses (see Table 2 for detailed results). First, decomposition of the omnibus effect of the *age of the faces* confirmed that adult faces were better categorized than child faces by all participants (Hypothesis 1). This difference explained 99.6% of the total variance of the *age of faces* effect, and the remaining unexplained variance (i.e., the residual treatment corresponding to 0.4% of the total between treatment sum of square for the main effect of the age of faces) was not significant, *F*(1, 96) = 1.12, *p* = .29, $\eta_{\text{p}}^{2}=.01$, showing that, among child faces, 7-year-old faces (*M* = .70, *SD* = .10) and 9-year-old faces (*M* = .72, *SD* = .13) were categorized in a similar way.

**Table 2.** Mean measures (A' and RTs), standard deviations, and statistics, for each tested hypothesis and its specific comparisons of interest.

Measure	Hypothesis	Prediction/comparisons	Mean (SD)	Statistic
***A'* scores**	**1**	*In all participants,* Adult faces > Child faces		
			.97 (.04)	*F*(1, 96) = 276.19, *p* < .001, η2p = .74
			.71 (.11)	

	2	Adult faces vs. Child faces, *in adults* > Adult faces vs. Child faces, *in children*	.98 (.02)	
			.74 (.12)	*F(1, 96) < 1, ns.*
			.96 (.04)	
			.70 (.10)	

	3	7-year-old faces* > * 9-year-old faces, *in 7-year-olds* 9-year-old faces* > * 7-year-old faces, *in 9-year-olds*	.69 (0.9)	
			.72(.13)	
			.68 (.10)	*F(1, 48) < 1, ns.*
			.71 (.11)	

**Reaction Times (in ms)**	**1**	*In all participants,* Adult faces < Child faces		
			1076 (299)	*F*(1, 96) = 276.19, *p* < .001, η2p = .74
			1293 (391)	

	2	Adult faces vs. Child faces, *in adults* > Adult faces vs. Child faces, *in children*	860 (184)	
			1321 (406)	*F(1, 96) < 1, ns.*
			1166 (293)	
			1281 (388)	

	3	7-year-old faces* < * 9-year-old faces, *in 7-year-olds* 9-year-old faces* < * 7-year-old faces, *in 9-year-olds*	1374 (347)	
			1347 (458)	*F(1, 48) < 1, ns.*
			1151 (341)	
			1251 (385)	

*ns* = not significant.

Concerning Hypotheses 2 and 3, contrast analysis did not reveal any kind of own-age advantage on accuracy. The magnitude of the adult bias (adult vs. child faces) in sex categorization of faces remained the same in child as in adult participants. Children (7- and 9-year-olds) were not better at categorizing the sex of their exact age-group relative to the other age-group, as predicted by a more specific own-age advantage.

### Response Bias

**Figure 3.**
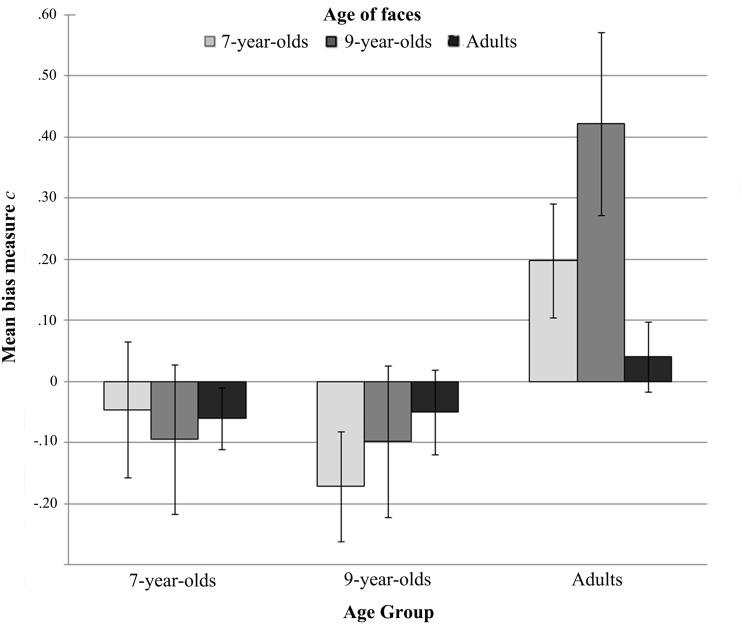
Mean response bias measure *C* (±*SE*s) for sex categorization of all age of faces for all participant age groups.

The response bias measure *C* (Figure 3) revealed a tendency to respond *female* more frequently than *male* in children, and the reverse in adults. One-sample *t* tests on *C* scores against the *no bias*' criterion (i.e., zero), separately performed for each combination of experimental condition and participant group, however, revealed that only the adult participants statistically respond more *male* when they categorized 9-year-old faces, *t*(14) = 2.83, *p* = .013, Cohen's *d* = .73, and tend to guess *male* when presented with 7-year-old faces, *t*(14) = 2.12, *p* = .052, Cohen's *d* = .55.

The response bias measures *C* were also analyzed using a two-way mixed ANOVA with *age of participants* (7-year-olds, 9-year-olds, and adults) as a between-subjects factor and *age of faces* (7-year-olds, 9-year-olds, and adults) as a within-subject factor. This analysis revealed only a significant main effect of the *age of participants, F*(2, 48) = 5.16, *p* = .009, $\eta_{\text{p}}^{2}=.18$. Adults showed stronger response bias (*M* = .22, *SD* = .43) compared with both 7-year-olds (*M* = −.07, *SD* = .41) and 9-year-olds (*M* = −.11, *SD* = .41), that did not differ from each other.

### Response Times

The RT measures correspond to participants' mean RTs for correct responses.

We ran a two-way mixed ANOVA with *age of participants* (7-year-olds, 9-year-olds, and adults) as a between-subjects factor and *age of faces* (7-year-olds, 9-year-olds, and adults) as a within-subject factor. The results can be seen in Figure 2. This analysis revealed a significant main effect of the *age of faces, F*(2, 96) = 17.76, *p* < .001, $\eta_{\text{p}}^{2}=.27$. More precisely, adult faces' RTs (*M* = 1,076 ms, *SD* = 299 ms) were smaller than both 9-year-old faces' RTs (*M* = 1,230 ms, *SD* = 394 ms) and 7-year-old faces' RTs (*M* = 1,354 ms, *SD* = 383 ms), that differed from each other (Post hoc Tukey Honestly Significant Difference [HSD]). The main effect of the *age of participants* was not significant, *F*(2, 48) = 1.74, *p* = .19, $\eta_{\text{p}}^{2}=.07$. Adults' reactions times were not faster than RTs of 7- and 9-year-old children, which did not differ from each other. There was a significant interaction between *age of faces* and *age of participants*, Greenhouse–Geisser adjusted *F*(3.39, 81.31) = 4.36, *p* = .005, $\eta_{\text{p}}^{2}=.15$. The interaction is further investigated in the following *a priori* contrasts (decomposition of the omnibus effects, see Table 2 for detailed results).

First, results confirmed that RTs for adult faces were shorter than RTs for child faces in all participants (Hypothesis 1). This difference explained 80.4% of the total variance of the *age of faces* effect. The test of the remaining unexplained variance was however significant, *F*(1, 96) = 6.96, *p* = .01, $\eta_{\text{p}}^{2}=.07$, showing that among child faces, RTs for 9-year-old were shorter than for 7-year-old faces.

Second, our results are consistent with an own-age advantage in adults, RTs for child faces increased compared with RTs for adult faces, whereas those RTs remained equivalent in child participants (Hypothesis 2). This contrast explained 78% of the total variance of the interaction, and the test of the remaining unexplained variance was not significant, *F*(3, 96) = 1.28, *p* = .29, $\eta_{\text{p}}^{2}=.04$.

Finally, there is no specific own-age advantage, as children categorized the sex of their exact age-group as quickly as the other age-group (Hypothesis 3).

In short, our experiment shows that both adults and children performed well at categorizing the sex of adult faces and that, whereas they succeeded in classifying the sex of child faces, their performance was poorer than for adult faces. Increased exposure to peers' faces does not seem to improve children's ability to determine the sex of a peer. We cannot however be sure that the age of the faces did not impacted sex perception. It seems, indeed, that age can modulate sex categorization. When participants are asked to categorize the sex of young and old faces, an asymmetry is observed. For young faces, the participants' performance is equivalent for both male and female faces. Female faces are, however, harder to categorize as female when face age increase, whereas male faces are categorized more easily with increasing age (Kloth, Damm, Schweinberger, & Wiese, 2015).

In our experiment, poorest performance for child faces could be due to a genuine difficulty in sex categorization for such faces. Children are indeed undergoing a period of transformation that might make their faces fundamentally difficult to ascribe sex to on the basis of their internal facial features alone. For example, mature faces tend to look more masculine (see e.g., Boothroyd et al., 2005) and girls start to mature earlier than boys do (Boxer, Tobin-Richards, & Petersen, 1983), the maturity of girls' faces may cause them to look as masculine as the less-mature boys' faces, resulting in reduced sexual dimorphism (difference between males and females) in children. During puberty, indeed, the rate of growth in head and face increases again around 9 to 10 years (Miklashevskaya, 1969), with earlier onset and greater growth in girls than in boys (Hautvast, 1971; Nellhaus 1968). Increased facial sexual dimorphism supposedly occurs around 12 to 14 years of age (Weston, Friday, & Lio, 2007).

To ascertain whether the age of faces affects the perception of sex, we asked adults to rate our stimuli on a masculine or feminine scale, in a supplementary experiment.

## Supplementary Experiment

### Participants

Twenty-eight independent adult observers (7 males; *M* age = 35.1, *SD* = 8.3) participated in the supplementary experiment.

### Procedure

Participants were asked to rate both the male and female pictures used in our experiment for each age-group on a 7-point scale in terms of how masculine the male faces were and how feminine the female faces were.

### Results

First, the degree of agreement between observers was high, as revealed by the intraclass correlation coefficient of 0.92 (95% confidence interval [0.89, 0.95]). Data were analyzed using an ANOVA with *sex* (male, female) and *age of faces* (adults, 7-year-olds, and 9-year-olds) as between subject factors on masculinity or femininity scores. In general, male faces were judged more masculine (*M* = 4.52, *SD* = 0.99) than female faces feminine (*M* = 4.05, *SD* = 0.96), *F*(1, 27) = 8.12, *p* = .008, $\eta_{\text{p}}^{2}=.23$. We found a significant main effect of the *age of faces, F*(1, 54) = 22.67, *p* < .001, $\eta_{\text{p}}^{2}=.46$. Post hoc Tukey HSD showed that adult faces (*M* = 4.84, *SD* = 0.90) had more exaggerated sex-specific facial traits than both 7-year-olds (*M* = 4.14, *SD* = 0.84) and 9-year-olds (*M* = 3.88, *SD* = 1.02; *p*s < .001), that did not differed from each other (*p* = .20). The *Sex × Age of Faces* interaction was also significant, *F*(1, 54) = 11.75, *p* < .001, $\eta_{\text{p}}^{2}=.30$. Post hoc Tukey HSD revealed that adult female faces were judged more feminine (*M* = 4.61, *SD* = 0.74) than both 7-year-old (*M* = 4.15, *SD* = 0.89; *p* < .001) and 9-year-old female faces (*M* = 3.38, *SD* = 0.82; *p* = .03). Nine-year-old-female faces were judged as the less feminine faces (*p*s<.001). In addition, adult male faces were judged more masculine (*M* = 5.08, *SD* = 1.0) than both 7-year-old (*M* = 4.13, *SD* = 0.79) and 9-year-old male faces (*M* = 4.37, *SD* = 0.96; *p*s<.001), that did not differ from each other (*p* = .56).

To sum up, the age of the faces seems to affect sex perception. Child faces are judged as less masculine or feminine than adult faces, which in fine result in a reduced sexual dimorphism for child faces, and particularly for 9-year-olds (as 9-year-old-female faces are perceived as more masculine than other female faces).

## General Discussion

The results confirm that adults and children performed well at categorizing the sex of adult faces in the absence of any gender-stereotypical cues. This is in line with previous literature, which showed that sex categorization is performed easily and accurately for adult faces (Burton et al., 1993; Wild et al., 2000). Although adults and children performance is nearly perfect for adult faces, it should be noted that children tend to respond more slowly than adults. Such age-related differences in processing speed are classically described in various tasks (e.g., Fry & Hale, 1996).

Participants were also less accurate at categorizing child faces (71%) than adult faces (97%), based on sex information. This is not surprising since repeated and continuous exposure to adult faces from birth is likely to offer a processing advantage for adult faces in both children and adults (see Macchi Cassia, 2011).

Importantly, in contrast to Wild et al.'s (2000) results, even the youngest children were able to categorize the sex of child faces above the chance level. Although accuracy did not reveal any influence of children's greater experience with child faces on sex categorization, which would manifest as a drop in magnitude of the observed adult bias, RT results are compatible with an own-age advantage in face processing. Adults were indeed slower to determine the sex of child faces compared with adult faces, whereas children's reaction times were similar for both type of faces, which may reflect a similar ease to process adult and child faces. Only adults seemed to adjust their RTs when exposed to child faces.

Our results showed that child faces elicited response biases in adults only. From our observations, it seems that children gave random responses when unsure, whereas adults might use a specific guessing strategy. As mentioned by Wild et al. (2000), there may be a social component to such bias, as a “higher price attached to mistaking a male for a female than for making the inverse error (p.289).” It is also possible that less familiar faces without salient diagnostic cues for sex, as distinctively female hairstyles, may be identified as *male*.

Overall, this pattern of results may be due to reduced sexual dimorphism in facial attributes in children. As confirmed by our supplementary experiment, age-related changes in faces influenced participants' perception of facial sex attributes. More specifically, sex-specific facial characteristics were less pronounced in children, and around 9 years of age, maturity causes girls to look less feminine. This support the hypothesis that a reduction in sexual dimorphism could impede the ability to categorize the sex of child faces.

In conclusion, whereas experience with a specific category of faces could result in advantage in speed of processing, difficulty in sex categorization of child faces could persist due to reduced sexual dimorphism in child faces. To further untangle the role of experience with faces and the effect of sex-specific facial characteristics, we suggest examining children and adults' ability to correctly categorize the sex of child and adult faces for which femininity and masculinity will be measure and degrees of sexual dimorphism manipulated.
